# Strengths-based practice in adult social care: Understanding implementation

**DOI:** 10.3310/nihropenres.13532.2

**Published:** 2024-08-22

**Authors:** Sharanya Mahesh, Ila Bharatan, Robin Miller

**Affiliations:** 1 Department of Social Work and Social Care, University of Birmingham, Birmingham, UK; 2 Warwick Business School, Coventry, UK

**Keywords:** strengths-based practice, adult social care, social work with adults, implementation

## Abstract

**Background:**

There has been increasing emphasis towards adopting strengths-based practice (SBP) within adult social care in England. Whilst there is agreement that SBP is the right approach to discharge adult social care duties, there is limited evidence regarding the implementation of SBP. This paper presents findings from the evaluation of the implementation of SBP in fourteen local authorities in one region in England.

**Methods:**

We employed a mixed methods research design, drawing on data from a scoping review, 36 interviews with practice leaders and two surveys, one with wider adult social care staff and the other, with external organisations like independent care providers and community and voluntary organisations. Our data collection and analysis were guided by two well established implementation theories: the Consolidated Framework for Implementation Research (CFIR) and Normalisation Process Theory (NPT). Interviews were analysed deductively, and surveys were analysed descriptively.

**Results:**

Local authorities are at different stages in their implementation journey. The Care Act 2014 and support for SBP demonstrated by key professional groups were seen as major drivers for implementing SBP. Whilst SBP resonated with the professional principles of social workers and occupational therapists, staff did not always have the confidence and skills to adapt to SBP. Changing paperwork and recording systems, providing training opportunities to develop staff competencies, establishing new care pathways, genuine co-production, and senior management buy-in were key enablers supporting implementation.

**Conclusions:**

To successfully implement SBP, a whole system approach that meaningfully collaborates with key professionals across sectors is essential. When implemented well, SBP has the potential to empower individuals by focusing on what matters to them.

## Introduction

Adult social care in England covers a broad spectrum of activities that provides personalised, practical support for people over 18 that are living with a disability or physical or mental illness to help them live independently stay safe and well ( The King's Fund, 2024). Support provided may be short-term or for a longer duration depending on individual circumstances. These can include support with daily living; reablement services to help regain independence; provision of aids and adaptations; support for family carers; and information and advice. Services can be provided in people’s homes, in day centres or in residential homes. The main legislation guiding adult social care in England is the Care Act 2014 which embeds the principles of strengths-based practice (SBP) Drawing on previously existing policies around ‘personalisation’, the Act takes a step further towards promoting wellbeing through historically and flexibly supporting individuals and their families. Whilst the Care Act does not explicitly require SBP as such, this term has subsequently been used by sector leaders to articulate the expected change in professional behaviours ( DoH, 2017) with a suite of resources from government and improvement bodies including practice frameworks ( DHSC, 2019) and related guidance ( RiPFA, 2019; SCIE, 2015).

There is no agreed definition of SBP within adult social care in the UK, and the term is used interchangeably with concepts such as ‘asset-based working’ (although for some bodies the first begins with individuals and the latter with communities (see e.g., SCIE & NICE, 2019; Caiels *et al*., 2021; Price *et al*., 2020). The most frequently used definition of SBP is cited by SCIE (2015), ‘ *Strengths-based practice is a collaborative process between the person supported by services and those supporting them, allowing them to work together to determine an outcome that draws on the person’s strength and assets. As such, it concerns itself principally with the quality of the relationship that develops between those providing and those being supported, as well as the elements that the person seeking support brings to the process’.*

Definitions of SBP have a common set of principles, including respectful and trust-based relationships, collaboration between individuals and professionals, focussing on what matters to the person, building on peoples’ informal and community networks, and positive management of risk ( Blood & Guthrie, 2018; DHSC, 2019; SCIE, 2015). Strengths have been classified as occurring on three domains – individual (qualities and beliefs of the person), interactional (engaging and collaborating with others), and contextual (society and communities) ( Janssen *et al*., 2011).

SBP is often described by what it is not, which is a ‘deficit-based’ approach that focusses on ‘what is wrong’, imposing solutions external to the person and their personal network, and working to pre-determined notions of success including reducing public expenditure ( Caiels *et al*., 2021; DHSC, 2019; Romeo, 2017). SBP in adult social care is not a tightly defined set of models and interventions, but rather a lose grouping of approaches which are based on common principles. In the UK, these include practice and development models working at an individual (e.g., family group conferencing, three conversations, solution focussed therapy), community level (e.g., local area coordination, asset-based community development), or system level (e.g., community led support, making safeguarding personal).

SBP is often connected with the profession of social work with academics at the University of Kansas being credited with its initial articulation in the early 1990’s (e.g., Saleebey, 2002; Weick *et al*., 1989) but also underpin professional standards of other professions (including occupational therapy and nursing) ( Gottlieb & Gottlieb, 2017; RCOT, 2019; Waterworth *et al*., 2020). There is though little direct research evidence to support its effectiveness in the UK context ( Price *et al*., 2020). This may be due to the inherent methodological challenge of researching preventative approaches, the diffused nature of its approaches, and a historic lack of funding for social care research in the UK in comparison with health care interventions ( Caiels *et al*., 2021; Price *et al*., 2020; Tew *et al*., 2019).

Research to date is clear that despite widespread support from much of the sector and its professions, fully embedding SBP is challenging ( Caiels *et al*., 2021; Price *et al*., 2020; Tew *et al*., 2019). This is of course not unique, with wider research on the implementation of the Care Act reporting that turning of legislation into practicalities was not fully achieved despite delivery being a particular focus for policy makers ( Burn & Needham, 2021). Challenges highlighted included context, clarity, complexity, collaboration, and capacity. SBP implementation difficulties are also not unique to adult social care ( Gottlieb & Gottlieb, 2017) and children social care ( Fenton *et al*., 2015).

This study is based on a formal evaluation that sought out to answer one question: how have local authorities in one region in England implemented strengths-based practice in adult social care. This region comprising of fourteen authorities offers a multifaceted context to study implementation since it comprises of urban and rural authorities as well as diversity in tailoring to economically and culturally diverse groups. In line with wider social care responsibilities in England, the local authorities in the region had autonomy to decide how to implement strengths-based practice within their local areas in order to meet their legal duties under the Care Act. Jointly undertaken by University of Birmingham and Warwick Business School, the study was funded by the National Institute of Health and Care Research’s (NIHR) Applied Research Collaboration West Midlands (ARC WM) and commissioned by the region’s Association of Directors of Adult Social Services (ADASS). The study was supported by Principal Social Workers (PSW) network, Occupational Therapists (OT) network and Commissioning (CC) network.

## Methodology

### Patient and public involvement

The study was guided by a panel of five people with lived experience who had either received adult social care services or had taken on a caring responsibility. The topic guide and survey questions were developed with the input of the lived experience advisory group. By providing feedback regarding interview and survey results, the group strengthened our interpretation of the findings.

### Research methods

In addition to the lived experience advisory group, this study was also guided by a stakeholder advisory group consisting of representatives from the regional DASS, PSW, LOT and CC networks. They too, were instrumental in offering guidance and support regrading recruitment of participants, framing research questions, developing topic guides and articulating findings.

We adopted a mixed methods research design that included a scoping review of evidence, interviews with practice leaders and surveys with adult social care staff and external organisations in the region ( Mahesh, 2024). A convergent mixed methods design supported with capturing diverse perspectives about implementation which was particularly helpful given the lack of evidence on implementation of SBP within adult social care ( Tew *et al*., 2019). Table 1 provides an overview of our methodology.

**Table 1.** Overview of the study.

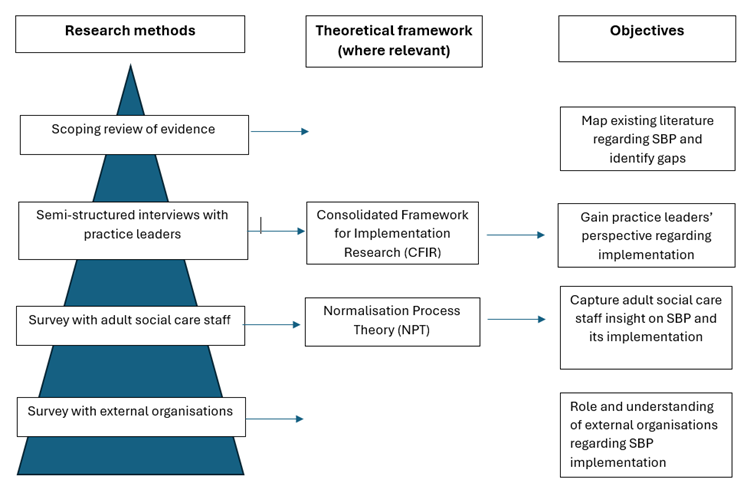

**Figure 1.**
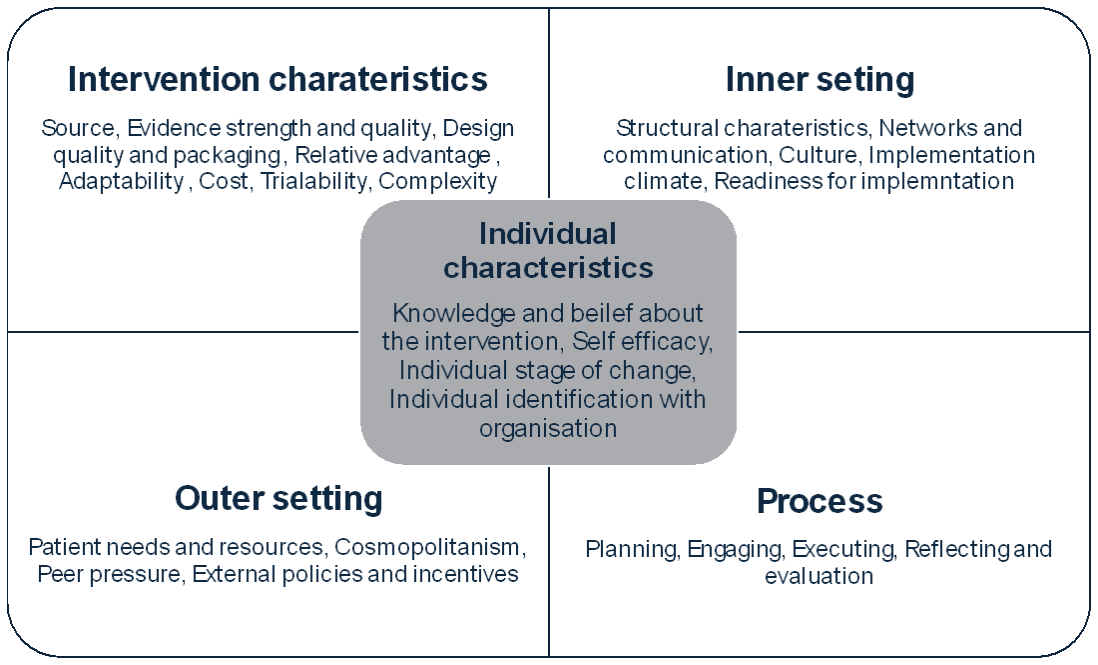
Consolidated Framework for Implementation Research (CFIR) constructs (Figure 1 has been partly reproduced with permission from Palinkas *et al.* (2015)).

Data collection and analysis were guided by two well established theoretical frameworks. For the interviews with practice leaders, we used the Consolidated Framework for Implementation Research (CFIR), a comprehensive framework that consists of a set of constructs that influence implementation within specific contexts ( Damschroder *et al*., 2009). CFIR was useful in examining changes introduced, common set of enablers and challenges across the five domains- intervention characteristics, outer setting, inner setting, individual characteristics, and process ( Figure 1).

Intervention characteristics mainly assess the rationale for implementing an approach, the adaptability and complexity of implementing within a specific context. Outer setting examines the external partners’ involvement and influence on implementation. The third domain, inner setting explores how internal factors such as organisational culture, processes and mechanisms, and leadership involvement’s impact on implementation. Characteristics of individuals focus on the influence that the interplay between individuals and their organisations have on their behaviour towards implementation. The final domain processes, examine the extent of planning, sub-processes and evaluations undertaken within the organisation to foster implementation ( Damschroder *et al*., 2009).

**Figure 2.**
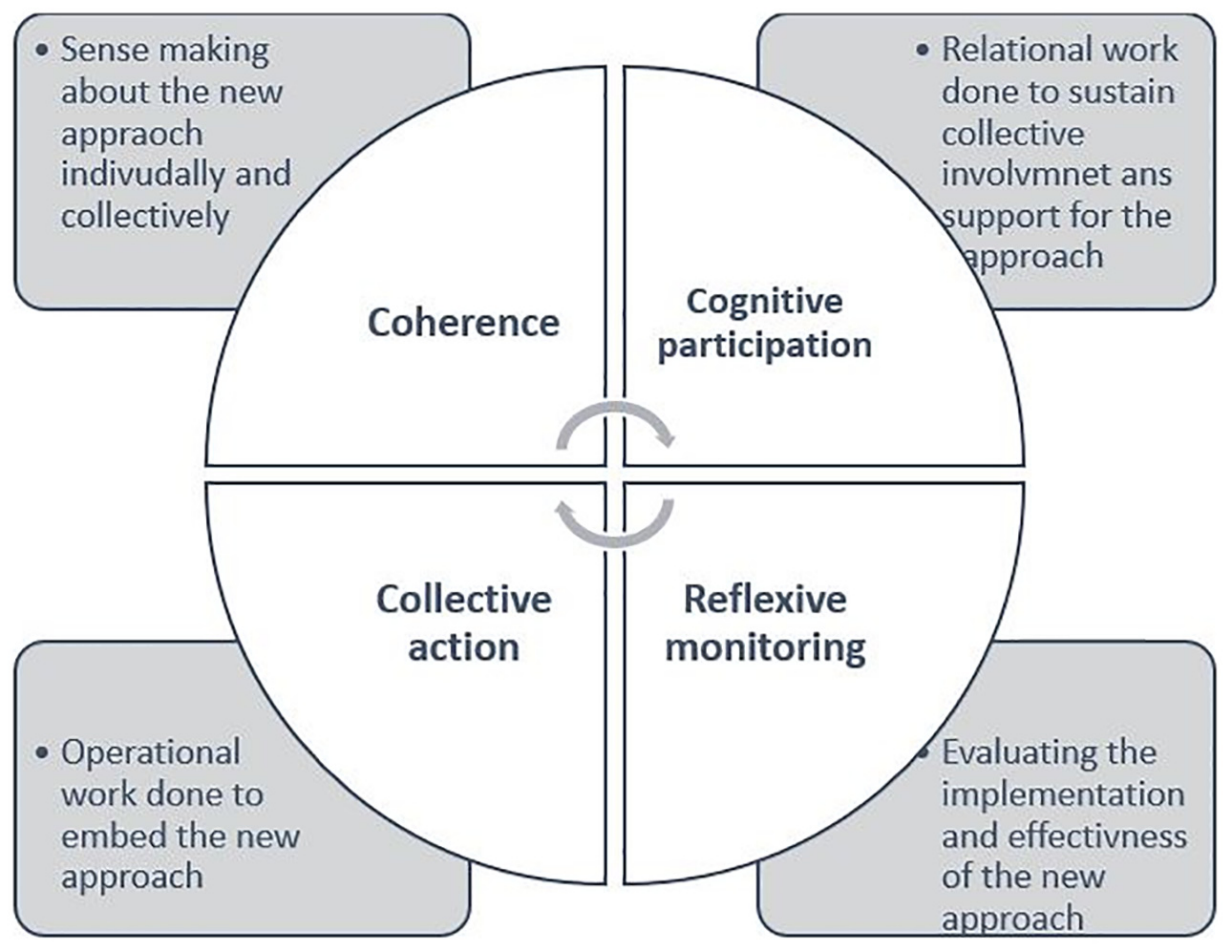
Normalisation Process Theory (NPT) framework (Figure 2 has been partly reproduced with permission from Huybrechts *et al.* (2023)).

We conducted two surveys, one with wider adult social care staff in the region and another, with external organisations including independent care providers and community, and voluntary sector organisations. Our survey involving adult social care staff was designed based on the Normalisation Process Theory (NPT) ( Figure 2). NPT serves as an explanatory framework that specifically focuses on change and perspectives of individuals directly involved in or affected by such change ( Schroeder *et al*., 2022). Combining both frameworks helped in systematic identification of the various components of implementation whilst allowing to capture diverse perspectives of different stakeholders.

### Data collection

We invited professional leaders i.e., Principal Social Worker (PSW), Lead Occupational Therapist (LOT) and Lead Care Commissioner (CC) from the fourteen local authorities and partnership organisations where social care was delegated to the NHS, to take part in an interview. Practice leaders from the above professions were invited to take part in the interview as they are viewed as the main professions within adult social care. Moreover, our aim was to gain a comprehensive understanding from leaders regarding the different strengths-based approaches introduced and their implementation processes. Between November 2020 and April 2021, 36 semi-structured interviews were conducted. The interviews took place on virtual platforms, were digitally recorded, and lasted for an average of 60 minutes ( Table 2).

**Table 2.** List of interviewees from each local authority.

Local Authority	Principal Social Worker (PSW)	Lead Occupational Therapist (OT)	Lead Commissioner (CC)
LA 1	×	×	×
LA 2	×	×	×
LA 3	×	×	×
LA 4		×	×
LA 5	×	×	
LA 6	×	×	
LA 7	×	×	×
LA 8	×	×	×
LA 9	×	×	×
LA 10	×	×	×
LA 11	×	×	×
LA 12	×		×
LA 13	×	×	
LA 14	×		×

The first survey with adult social care staff was developed to understand perspectives of all staff working in or supporting adult social care service provision and delivery and was developed partly based on the initial analysis of the interview data. We received 286 responses, and our participants mainly included social workers and occupational therapists. We also received responses from other professionals such as commissioners, housing and finance staff, in-house care providers and case workers. A majority of our respondents described themselves as currently having frontline responsibilities with about a fifth each reporting that they supported older people and people with physical disabilities. Most survey respondents reported having over five years of experience not only within adult social care but also within their current local authority. The survey included questions about motivation and involvement of staff towards SBP and its implementation, support available and organisational conditions within which SBP was implemented. The distribution of this survey was facilitated by the regional PSW network.

Our second survey with external organisations was aimed at gaining the perspectives of independent care providers and community and voluntary sector organisations regarding SBP, their involvement in supporting local authorities in becoming more strengths-based and visa-versa and, resources necessary to embed SBP across the system. This survey was distributed through the lead commissioners to all organisations which they viewed as supporting delivery of adult social care. Of the 131 responses, a majority of them reported as holding senior or middle level managerial positions and having more five years of experience within their services. Our respondents supported a variety of different populations including older people, individuals with physical difficulties, learning disabilities and people with mental health difficulties. Services offered included residential care and/or domiciliary, day care and providing information and advice. Both surveys were piloted with our stakeholder advisory group which led to minor modifications in language and order of questions.

Surveys were developed using the Qualtrics XM survey tool. Whilst the study employed a paid version of the survey tool, a free version is available to use which offers similar features for replication of our surveys. For survey response options, we used Likert-type responses ranging from ‘strongly agree’ to ‘strongly disagree’ with the central option attributed to ‘neutral’ suggesting an equal distance between each option ( Bishop & Herron, 2015). Likert scales are particularly useful to quantify and evaluate the perceptions and attitudes of respondents which fit sufficiently well with our intended purpose of conducting the surveys.

### Data analysis

We conducted deductive thematic analysis for the qualitative components of the study namely, the interviews and open-ended questions in the surveys. Deductive thematic analysis or theoretical driven coding uses theory as its points of departure ( Boyatzis, 1998). Interviews were mapped against the CFIR constructs. Prior to undertaking analysis, the research team examined the CFIR constructs, leading to selecting constructs that were relevant to the study. For example, our analysis excluded the domain on the individual characteristics, as our aim was to study implementation at a strategic level rather than at the individual level. Our survey data was analysed descriptively. Mainly, we measured frequency distribution of our data sets to capture overall perceptions of adult social care staff and external organisations. Graphs were generated using the survey tools analytical functions.

By adopting a convergent mixed methods design, we able to triangulate our data from the interviews and particularly, the adult social care staff survey. Upon analysis of our interview findings, our survey data was mapped against these findings to determine any congruence or disparities in opinions among our participants. In doing so, we were also able to identify new themes that emerged in one of the two data sets and examine its implications.

### Ethics

Approval for the study was obtained from the University of Birmingham’s ethics committee, approval numbers ERN_20-1163 (approved on 23.10.2020) and ERN_21-1151 (approved on 20.08.2021). All participants have either provided verbal and/or written informed consent to take part in the research. In accordance with approval from the University’s ethics committee, where participants did not provide prior written consent, verbal recorded consent was obtained at the start of the interview following the participant information sheet being read out.

## Results

### Rationale and approach for introducing SBP (Intervention characteristics)

The implementation of SBP was notably influenced by the Care Act 2014 although, at least two local authorities had implemented one or more SB approach prior to the policy. Alongside the Care Act 2014, the principles of SBP aligned with the professional principles and values of social workers (SW) and occupational therapists (OT) who strongly emphasised that SBP was ‘morally the right thing to do’. Furthermore, OTs consistently reported that SBP was consistent with their existing professional standards and ethics, suggesting that they always worked in a strengths-based way although, the terminology may have varied. For some local authorities, SBP was seen to have the potential to create resource efficiencies through reducing reliance of long-term and intensive health and care services thereby, acting as an additional driver to undertake implementation.

***“It’s about doing the right thing and it’s about better outcomes for the people of XXX and that’s very much where our director is” (PSW 11).***

***“I think generally the motivation is a sense of morality in some ways, is that we all know ourselves that if we’re struggling with something we learn best by other people around us, either learning ourselves how to go about doing that task that we had” (OT 13)***

**Figure 3.**
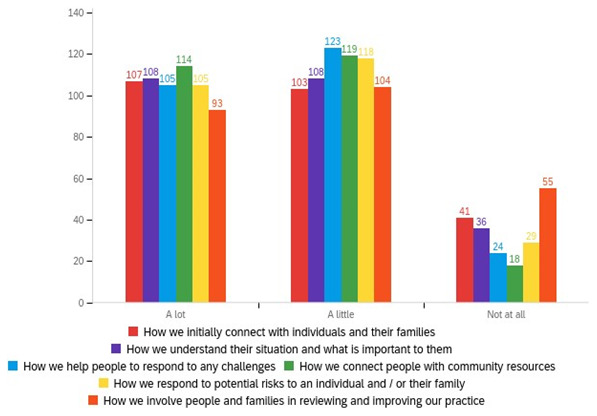
Perceptions from adult social care staff in the local authority regarding the extent of change in practice to support SBP.

Whilst our interviewees stressed that the principles of SBP resonated with the underlying principles of main professional groups, it appears that local authorities as organisations did not always reflect strengths-based principles in their processes. Common examples shared included deficit-based paperwork and recording systems that were lengthy and didn’t encourage strengths-based conversations limited or constrained care delivery pathways that usually had a single front door and lengthy waiting periods and, a focus on performance management all of which were seen to prevent practice from being strengths-based. The extent of these limiting processes was reflected in the proportion of changes being introduced by local authorities ( Figure 3). Over 48% of respondents to the adult social care staff survey reported that ‘a lot’ has changed in the way they connect people to community resources. Just over 42% of respondents also reported that ‘a lot’ has changed with how they initially connect with individuals and families and, recognising what is important to individuals.

The decision on which SB approach (s) to implement appeared to be influenced by a few factors. Largely, it appeared to be led on the recommendations made by the PSWs (who were given the lead or key role in implementation) which were based on their assessment of the practice conditions, available resources and consideration of what may be beneficial to their organisation. Furthermore, approaches adopted by other organisations in the region and elsewhere in the country appeared to influence which SBP approach was implemented. Decisions were rarely based on formal research evidence which may be in part due to a perception that there was a lack of related research and where evidence was available, this was not always readily available to local authorities.

Local authorities had different views of the various models, which may be due to the lack of formal research evidence. An example of such an approach is the Three Conversations model (3Cs). 3Cs is a set of principles that support practitioners to have open conversations with individuals and their families that focus on strengths and desires whilst connecting them with appropriate resources to improve their wellbeing. Some local authorities implemented 3Cs with the support from Partners 4 Change whilst some other local authorities implemented an adapted version of 3Cs, partly due to the actual model not being seen as entirely relevant to the local context as well as the lack of funding available to commission Partners 4 Change. Particularly, a few local authorities reported that the approach as endorsed by Partners4Change had somewhat become a tick-box exercise and formulaic, diluting the principles of SBP. However, irrespective of the model implemented, our interviews did not highlight any substantial advantage of adopting 3Cs when considered against other SB approaches. This was based on the perception that 3Cs was among many other strengths-based approaches that was available. But in a few local authorities, positive outcomes reported by local evaluations elsewhere influenced their decision to implement 3Cs.

***“I had done a bit of research around the different approaches that different local authorities had used. With three C’s, there were a couple of authorities at the time who were categorically telling us that there were some definite differences, so they were able to” (PSW 13)***

***“I have got issues with the three-conversation model. In that three-conversation model says it’s not about assessing, it’s not about, it’s about conversation with somebody, just because you don’t say the word, doesn’t mean you're not doing assessments” (PSW 8)***

Implementation of SBP appears to have been undertaken in phases, with many local authorities conducting pilots, before a full rollout although, in most cases, pilots were conducted to manage resources and extent of change to be introduced all at once rather than with the intention to test and modify implementation before a full rollout. In most local authorities, there was a narrow scope for the implementation of SBP, mainly focusing on changing care planning within social work practice, with little evidence of support provided to other professionals and external organisations to implement SBP within their own practice. Findings from the survey with external organisations corroborated our interview findings as only 28% respondents ‘strongly agreed’ or ‘agreed’ that their service was informed about the local authority seeking to be more strengths-based in their work.

***“We have a pilot running in one of our locality teams to work using these strength-based tools. So, we’ve taken a review of that, in fact we reviewed it back in May and we did some large-scale auditing and that informed our transformation plan.” (PSW 11)***

### External influence on implementation (Outer setting)

Austerity was paradoxically both a driver and barrier for SBP. There was an expectation that SBP would lead to much needed service efficiencies and potentially savings through reducing demand for longer term support while on the other hand, austerity appeared to limit the amount of resources at their disposal to allocate towards implementation.

***“I think there’s always been a tension in terms of what you can do. Because we’ve been doing this with all the sort of austerity going on at the same time, we’ve always been constrained in terms of what spend there can be. You can want to be strength based and promoting somebody who’s independent, but you had to do it at best value”. (PSW 14)***

**Figure 4.**
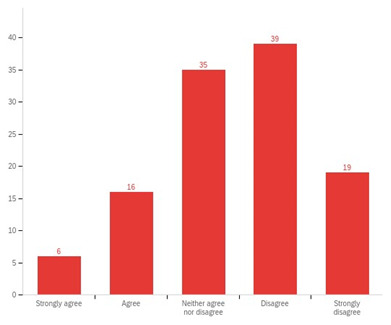
Perceptions of external organisations regarding their involvement in their local authority’s aspirations to be more strengths-based.

Our interviewees reported that the involvement and support of external organisations like independent care providers and community and voluntary organisations was essential to embed SBP across the system. However, over 50% of respondents to the external organisations survey ‘strongly disagreed’ or ‘disagreed’ that their organisations were consulted and/or involved in how their local authority was seeking to be more strengths-based. An additional 30% respondents ‘neither agreed nor disagreed’ of their involvement to develop SBP in their local region ( Figure 4). Furthermore, over 75% respondents reported that they were not provided with adequate resources and support from their local authority. In addition to funding, more information and training opportunities were reported as essential for external organisations to embed SBP. It appears that although some local authorities may have included health services within their implementation plans, health services were not seen to be collaborating with SBP programmes and open to such social care led developments. This tension appeared to have stemmed from different practice principles, with health services reported to be prescriptive and social care operating from a more preventative and early intervention approach.

***“Some can be a little more old school than others and the difficulty I find professionally with people working in healthcare is that they tend to be more of a medical model approach to intervention rather than a social model” (OT 07)***

Local authorities were compelled to work in a more collaborative way during the pandemic, positively impacting the relationship between local authorities and external organisations. Setting up of new processes reflecting the principles of SBP, as well as developing strong ties in the community, appeared to support the implementation of SBP not just within the local authority but also encouraged external organisations to adapt SBP in their organisation. At the regional level, support from their regional ADASS and practice networks facilitated strong relationships between local authorities alongside providing them a platform to share experiences, ideas and learn from others about their implementation journeys.

***“I think we’ve got a much better relationship with our providers and again, giving the example of the day service providers, it’s sort of really paid off and we’ve seen how it’s worked during Covid. We’ve been in touch with them very, very closely and working together in partnership to sort of make things work better” (CC 14)***

While SBP prioritises individuals placing them at the centre, we noted limited evidence of consistently involving individuals and families during the initial stages of SBP design and implementation. It appeared that implementation was largely professionally led, often without a clear intention or plans for engaging with individuals with lived experience. Furthermore, there was little evidence to suggest that individuals and their families were informed about the new approaches introduced or new ways of working. Despite local authorities consistently reporting their priorities towards individuals with lived experience, we noted only pockets of engagement and consultation activities. In some authorities, consultations were held about specific activities relating to SBP while in a few authorities, a greater degree of involvement was noted, with individuals co-producing some care pathways, giving people ownership, and improving the chance for successful implementation.

***“I think just one of the areas that is really kind of coming into its own is working together with people. So, the experience through the last year with the direct payment work of working in a coproduced way is as just being incredible, it’s been time consuming though. We’ve got people who use direct payments now who feel that they are they are totally part of this thing and we’re all in it together, and we are human beings” (PSW 06)***

Our interviewees also reported that individuals with lived experience and their families could seem reluctant to engage with a more SB approach. In particular, such resistance was demonstrated by individuals who had multiple complexities of need or, where an established package of support was already in place. Particularly for people experiencing multiple complexities, there was a notion that talking about ‘what they can do’ was not entirely appropriate atleast during initial stages of engagement. Individuals approach services due to a deficit and in some cases, with multiple challenges, making it difficult for practitioners to encourage them to clearly see their strengths or instil aspiration. There appeared at times to be tension between expectations of the individual and/or their family and the local authority’s perception on the most beneficial type of care. Whilst local authorities were keen to explore resources available within the family network and community to support individuals, individuals did not always understand its purpose and feared that their care packages may be removed or reduced, resulting in disengagement with services. The limited participation of individuals in the implementation process and minimal sharing of information may have contributed to people perceiving SBP as professionals shifting their responsibilities onto individuals. Moreover, feedback from the study’s lived experience advisory group highlighted that most individuals were not familiar with the term SBP and were unaware that local authorities were working towards being more strengths-based, which may in part be due to the lack of trust that individuals had on their local authorities in improving their wellbeing.

***“So, we’re seeing more and more complex people coming to us, so it can make it challenging to focus on those strengths when there are a lot of challenges for those individuals”. (CC 06)***

### Organisational factors influencing implementation (Inner setting)

Structural change was a common approach to embedding SBP, notably restructuring of their social work teams to be more aligned with the local requirements for offering support. There was generally emphasis on the benefit of multidisciplinary working, specifically encouraging SWs and OTs to work more collaboratively and bring their different skills sets togethers. However, this perception was challenged in a few local authorities, who questioned the extent to which SWs and OTs working together would positively impact embedding SBP.

***“I have very little contact with my social work colleagues. However, as part of being team manager in social care, you're on a rota panel and you're reading assessments which are about asking for approval for residential nursing care or home care. I haven't seen that much difference” (OT 03)***

SBP appeared to drive the relocation of certain SW and other services like occupational therapy into the community. Relocating services was generally a part of the local authorities’ implementation plans, an approach that appeared to support them with their SBP agenda. Moving some services into the community appeared to present an opportunity to develop relationships with partner organisations and facilitate access to community resources.

***“Huddles [community lounges] are definitely the best thing that’s happened, the teams love them, and you know we’ve had health involved, housing officers have made a difference in that, bringing expertise in to help support those decisions so then people aren’t kind of handed over to loads of different people” (PSW 13)***

Alongside encouraging multi-disciplinary working between SWs and OTs, emphasis was given to developing and enhancing connections within social care divisions and with other functions in the local authority. For instance, social workers increasingly reported developing rapport with commissioning and housing staff, to enable joined up working between services with the aim to embed SBP across the local authority.

While SBP theoretically aligned with the principles of SW and OT practice, it appeared that both professions did not necessarily work in a strengths-based way, with some staff demonstrating reluctance to adapt to SBP. In a few local authorities, the introduction of SBP appeared to increase pre-existing tensions between SWs and OTs, with SWs questioning the increasing role, presence, and input from OTs. We noted from OTs, that they may have experienced a degree of marginalisation as social workers were predominantly dictating the direction of OT services. This was despite OTs reporting that they had been working this way even before SW adapted SBP. Social care providers and the VCS were more positive with 72% of respondents to the external organisation survey ‘strongly agreeing’ or ‘agreeing’ that their staff were positive about working in a strengths-based way.

***“It’s been hard work because obviously some of the social workers felt, not threatened, but a little bit ‘well I’ve been doing it this way for years, I’m quite capable of making a decision, why do I need an OT to tell me whether or not I’m allowed to make that decision’” (OT 02)***

Our survey with adult social care staff highlighted that over 75% of respondents ‘strongly agreed’ or ‘agreed’ that they felt confident in their skills to demonstrate SBP. However, our interviewees reported that social workers did not always have the confidence and skills to work in a strengths-based way. It appeared that SWs had become accustomed to using lengthy forms and deficit-based questioning which created challenges to adapting to new ways of working that required them to think creatively and engage in positive risk taking. This shift in approach appeared to cause a degree of anxiety for some staff however, where staff were more actively involved in decision making regarding implementation, we noted more openness and confidence from staff. Moreover, where training opportunities were provided, it appeared to enhance the confidence and skills of staff.

***“Well, it’s culture change isn’t it and in culture change it’s hard, it’s difficult, some people can’t wait to do things differently and some people find it more difficult to move away from what they have been doing so transition takes time. You have to keep working at it” (PSW 13)***

**Figure 5.**
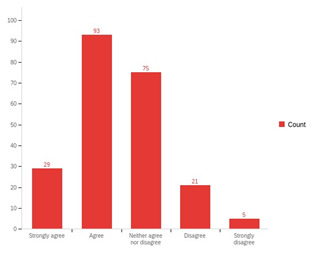
Perceptions of adult social care staff regarding supportive organisational culture in their local authority.

Despite some tensions raised regarding staff reluctance during the initial phases of implementation, over 95% of adult social care staff respondents ‘strongly agreed’ or ‘agreed’ that they were open to SBP. However, just under 50% of respondents reported that their organisational culture did not support SBP suggesting that organisational and practice conditions were not always supportive of SBP ( Figure 5).

Whilst there appeared to be no well-defined and structured implementation plans, local authorities steadily introduced changes that improved the implementation climate (i.e., how well adult social care divisions can initiate and handle change) to support embedding SBP. In addition to structural changes, organisations have introduced changes to paperwork and recording systems, care pathways that enabled flexible working between professional groups and easy access to services in the community, developed relationships with external organisations and updated supervision and training policies. Over 50% of external organisations survey respondents reported that they needed similar support such as, changing IT systems, reorganising roles and responsibilities of existing staff and working more closely with other organisations to implement SBP within their own organisation.

***“We redesigned the customer’s journey, we redesigned the entire adult social care pathway, we redesigned the social care training programme. In fact, we came up with quite some innovative ways of training our staff around strengths-based practice. That journey really started there, and there was a huge investment in terms of practice and culture change” (PSW 04)***

The extent of undertaking these initiatives appeared to largely depend on the level of support from senior leadership and resources made available for implementation. In local authorities where there was buy-in from senior management, the director of adult social services was central to setting the vision, actively participating in developing implementation plans and working closely with practice leaders (most notably PSW, SB lead) to execute these plans. Moreover, when senior leaders made themselves accessible to frontline staff in the implementation process, it appeared to demonstrate the organisation’s commitment and help develop a receptive culture. Overall, where senior leadership was involved, local authorities appeared to have made most progress with embedding SBP.

***“I’d say we have got an incredibly supportive director and I think he had a vision, and he built his management team around his requirements and observations of the local community. I think it’s absolutely from the top down, but equally from the bottom up because what we’ve put is real emphasis on the voice of the practitioner” (PSW 03)***

The level of involvement of senior management appeared to correlate with the amount and type of resources made available for implementation. Where there was greater degree of involvement of senior leaders, we noted local authorities deploying financial resources and employing additional staff to support with implementation. Moreover, we noted increased training opportunities for staff at all levels to develop leadership skills and to increase their confidence to adapt to SBP. In some local authorities, austerity, and the lack of support from senior leadership resulted in limited resources deployed for implementation, which were mainly drawn from a pool of existing resources.

***“A good amount of money was made available and yes, we had a dedicated project officer and for most of the programme a dedicated project support officer as well. The rest of the group added it, fitted it in with our day jobs with the work we were already doing (PSW 13)***

### Implementation process (Process)

It appears that there were rarely explicit plans for the implementation of SBP and it proved surprisingly difficult to obtain up-to-date detailed and concrete plans from most local authorities. In some cases, this was because SBP implementation was part of wider transformations undertaken within the local authority. Where plans were available, these tended to be focused on specific activities or projects such as training or supervision policy or development or modification of care pathways that were initiated to support SBP rather than a whole-system transformation. Even in such focused examples, plans were not detailed. 

Given the narrowed vision for SBP, mainly related to SW practice, we noted limited engagement of other professional groups in implementation including occupational therapists. Where other professional groups and external organisations were involved, we noted that local authorities were able to draw from a larger pool of expertise about SBP whilst being able to accommodate practice requirements of other professions thereby garnering support and increasing the chances for successful implementation.

***“With the two pilots, we had a project team, so we had quite a lot of investment from the council itself. So, we had a project team and crucially in both projects it was our director of adult services who was the lead. And then obviously we had the senior management team, and we had finance, and we had insight and performance” (OT 13)***

***“Well, if you're not in mainstream social work, in adult social care, you tend to be a sort of afterthought when anything is introduced” (OT 07)***

**Figure 6.**
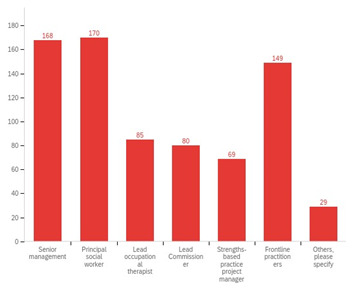
Perceptions of local authority adult social care staff regarding the persons responsible for leading implementation in their organisation.

PSWs and senior management were commonly identified as the main leaders for leading implementation of SBP ( Figure 6). Notably, other practice leaders such as LOTs and CC, integral within adult social care, were reported to have limited involvement. Moreover, just over 50% of respondents to adult social care staff survey ‘strongly agreed’ or ‘agreed’ that they were able to contribute to the development of SBP in their respective local authorities, suggesting the involvement of only a select few within implementation.

A common approach to supporting implementation was training SBP champions. We noted a degree of inconsistency in their roles and responsibilities of champions, but their overall purpose appeared to be two-fold (1) act as an SBP expert in the local authority and provide practical support to other staff (2) support with implementation activities- establishing processes and engaging with partners.

***“Because it was a new role, we had to think about what was expected of the operational lead and it was primarily to work with staff across the adult care teams, the community teams and with our commissioning and brokerage service to launch strengths-based practice” (SBP lead 07)***

In a few local authorities, external change agents like consultancies were contracted to drive implementation. There was variation as to at which points in the implementation of SBP they were employed, but they were generally contracted to help support with the implementation of specific SB approach. Support was mainly provided with setting up systems, sharing information, training staff, and measuring outcomes in some instances. Where local authorities worked with consultancies, this appeared to help provide a more coherent structure for implementation (atleast during the initial stages). Their support included encouraging authorities to develop a clear plan with timelines, articulate necessary steps and resources required to initiate change, offer additional support and guidance during implementation and, undertake reviews to check implementation progress. However, the processes which were developed were not always sustained once consultancies had withdrawn.

***“We had support from XXX and we tried it out in a couple of teams, looked at systems and bureaucracy, stripping it all back, just trying to reduce people being handed over a lot and triaging and working with the teams closely, looking at you know help supporting them through that change. We did several evaluations, and we evolved the innovation sites over time” (PSW 13)***

To implement SBP, we noted a common set of enablers although, there was considerable variation in the blend and extent of these activities. Common enablers included- developing and/or revising existing care pathways, training, and reflective supervision to develop competencies of staff, establishing SBP supportive processes and frameworks such as new recording systems, changing assessment forms and developing quality assurance frameworks, and building relationships and collaborating with external partners.

***“We’ve got three work streams up and running now. So, the first one is around the performance and the systems, so that’s kind of the end-to-end process. The second work stream is around learning development and what skills the workforce needs and how we can make sure that we’ve got a good offer around that. And the third work stream is around connecting with communities” (PSW 11)***

The degree to which local authorities have been successful in the implementation of SBP is unclear. Whilst a few local authorities had established baseline indicators, there was limited evidence of any evaluations undertaken. Most local authorities reported that establishing measurement indicators and undertaking evaluations was a part of the next phase of embedding SBP however, these indicators appeared to focus more on measuring effectiveness of SBP and less on evaluating implementation processes. Nevertheless, periodic audits (in some local authorities) and practice reviews undertaken as part of the regional ADASS’s peer challenge process appeared to provide helpful insights about SBP.

## Discussion

The aim of this study was to examine the implementation of SBP in fourteen local authorities in one region in England. Recent reviews have articulated the gap in research evidence ( Price *et al*., 2020) and challenges to study strengths-based approaches in practice ( Caiels *et al*., 2021). This study seeks to fill these notable gaps by systematically studying the implementation of SBP in adult social work and social care. Our findings suggest that the Care Act 2014 laid an important foundation for local authorities and external organisations to implement SBP. This widespread support for SBP from much of the sector and its professions ( Tew *et al*., 2019) may be partly explained by the resonance between the principles of SBP and the professional values and principles of social workers and occupational therapists. Moreover, social workers and occupational therapists viewing SBP as ‘morally the right thing to do’ was seen as an additional driver for implementation. However, the need for local authorities to implement a number of changes to support implementation suggest that although in principle social workers and occupational therapists aspired to be strengths-based, it did not necessarily translate into practical application in their work with individuals and families.

Implementation of SBP is likely to be more successful when leadership at all levels actively participate but buy-in from senior leadership was particularly crucial ( Duggal *et al*., 2021). A common issue when implementing any new approach are the inconsistencies in value systems of leaders ( Lunt *et al*., 2020; Wildman *et al*., 2019) which may increase the likelihood of a failed implementation. However, our study highlighted that when a top-down approach was taken, it created an opportunity for senior managers to set a clear vision and sustain leadership involvement during and after implementation. Moreover, where senior managers were actively involved, we noted more training opportunities to develop leadership competencies of staff at all levels, which positively supported team leaders and staff by partly mitigating their anxieties and equipping them with the skills and confidence to support implementation ( Cooper *et al*., 2018; Hamilton *et al*., 2015). However, as with any top-down approach, some staff felt disengaged ( Cooper *et al.*, 2018), impacting their level of support for SBP.

Linked to staff disengagement, our study highlighted some degree of reluctance from staff to adapt to SBP. Staff training was useful to support implementation ( Butler & Manthorpe, 2016; Symonds *et al.,* 2019) but these were more effective when introduced alongside other initiatives such as peer support, reflective supervision and changing recording systems ( Butler & Manthorpe, 2016; Symonds *et al*., 2019; Velzke, 2017). Training targeted to support implementation of specific strengths-based approach, appeared to be more effective due to the clarity regarding principles and processes inherent to that approach.

During the initial stages of implementation, there was an assumption that SBP was limited to social work practice, limiting collaboration with and involvement of other professional groups (example OTs). Pre-existing divisions and boundaries between SWs and OTs, with each professional group trying to uphold their independence ( Abbott, 1988) may have further contributed to the direction taken for implementation (mainly directed to modifying social work practice) alongside increasing resistance from professionals to support implementation when not adequately involved ( Abbott, 1988). Working with other professional groups provides a real opportunity to develop a shared vision ( Kaehne, 2020), draw on expertise of others and embed systems and processes acceptable to all professionals. Moreover, by involving other professional groups, there is reduced risk of them feeling excluded and therefore are more supportive to the innovation whilst increasing the likelihood of positive engagement.

For SBP to be fully embedded and sustained, a whole system approach is crucial, which can be achieved by involving relevant external stakeholders. Despite studies consistently reporting the significance of gaining support ( Cooper *et al.*, 2018; Hamilton *et al*., 2015) and collaborating ( Baron & Stanley, 2019) with external organisations, the restricted notion of SBP appeared to limit involvement and engagement with external organisations. Although local authority staff highlighted that investing in and working with the community and voluntary sector are crucial for SBP, this was not always reflected in the resources and support offered by local authorities. Lack of engagement implied that SBP was an internal innovation but equally, limited the opportunity to encourage external organisations to adopt SBP within their own organisations. Having said that, mapping community resources, relocating services into the community and, the pandemic appeared to strengthen relationships with external organisations.

Moreover, involvement of people with lived experience was limited during SBP design and implementation. Co-production, though central within policy (Care Act 2014) and practice ( CFE, 2020), our findings suggested a significant degree of variation in the nature and extent of co-production activities carried out in local authorities. Where voices of people with lived experience were included, services were able to contribute towards identifying needs of people ( Darley & Carroll, 2022), recognise barriers in service delivery and encourage innovation ( Romm *et al*., 2019). To fully involve individuals and families, local authorities must share adequate information about their plans and invest in organising relevant documents such as handbooks with easy read options to help individuals and families understand the purpose of SBP and its potential impacts.

More generally, the lack of data remains a challenge within adult social care ( Cream *et al*., 2022) and particularly, in relation to understanding impacts of innovations. Where local authorities reviewed their pilots, it provided useful insights regarding processes and systems alongside shedding light on the positives and challenges to implementation ( Ettelt *et al*., 2013). Broadly, the lack of concrete plans for implementation alongside no clear measurement indicators for both implementation and effectiveness elicited a serious challenge to determine the extent to which SBP had been successfully implemented.

## Conclusions

SBP continues to benefit from widespread support from both, the sector and policymakers, suggesting its central role for fulfilling adult social care duties. However, little is known about the implementation and effectiveness of SBP which is largely because SBP represents a set of principles that may be subject to varied interpretations within and between professions. When implemented well, SBP has the potential to empower and improve wellbeing of individuals by focusing on their strengths and what matters to them. To increase the likelihood of sustainable implementation, organisations must deploy a whole system approach that meaningfully involves all partners and stakeholders in adult social care. This study highlights the importance of systematically studying implementation. More research needs to be undertaken to study both, implementation, and effectiveness of SBP as with the lack of such evidence, it increases the risk of local authorities continuing to implement SBP without fully understanding its impacts. 

## Consent

Verbal and/or written informed consent for publication of the participants’ details was obtained from the participants.

## Data Availability

Our qualitative data which includes semi-structured interviews with practice leaders from a specific geographical region makes it challenging to de-identify data at the individual level to an acceptable standard. To request access to restricted data, please contact Sharanya Mahesh: s.n.mahesh@bham.ac.uk Harvard Dataverse: Extended data for ‘Strengths-based practice in adult social care: Understanding implementation’, https://doi.org/10.7910/DVN/RTHIIF ( Mahesh, 2024) This project contains the following underlying data: External organisation survey.pdf Local authority staff survey.pdf Data are available under the terms of the Creative Commons Zero “No rights reserved” data waiver (CC0 1.0 Public domain dedication).
