# Supplementary material for: Strengths-based practice in adult social care: Understanding implementation
Source: NIHR Open Res. 2024 Aug 22;4:19. Originally published 2024 Apr 16. [Version 2] doi: 10.3310/nihropenres.13532.2 (PMC11399757; doi:10.3310/nihropenres.13532.2)
Supplement: Peer review history

# Reviewer report: Reviewer response for version 2

**DOI:** 10.3310/nihropenres.14903.r32740

**Published:** 2024-09-30

**Authors:** TJ Robinson Moncatar

**Affiliations:** 1 Department of Health Policy and Administration, College of Public Health, University of the Philippines Manila, Manila, Metro Manila, Philippines

The revised article is interesting and well-written. However, there are a few aspects of the paper that the author may further improve:

 1. In the abstract under methods, the authors may want to be more specific with the mixed methods design (convergent-parallel) used in the study. The author may also want to consider to further elaborate the use of this design in the main methods section as well.

 2. In the introduction section, the authors may describe the research gap to justify on why such research study is being conducted can still be strengthened. Aside from such assessment is not yet conducted, what is happening as of the moment that warrants assessment in such a way that the evidences that will be derived will assist in redesigning of the intervention implemented.

 3. The relevance of this study can be improved. The authors may further elaborate on how other context would benefit from the results of the study.

 4. The study objectives can be clarified or highlighted after the research gap and before the study significance. Is it focused on how it is being implement and what are the intended and unintended outcomes (effectiveness or fidelity)? 

 5. In the discussion, it would be critical to compare for similarities and differences of the study results with other published articles.

 6. Under discussion section, add a strengths and limitation.

Is the work clearly and accurately presented and does it cite the current literature?

Partly

If applicable, is the statistical analysis and its interpretation appropriate?

Yes

Are all the source data underlying the results available to ensure full reproducibility?

Yes

Is the study design appropriate and is the work technically sound?

Yes

Are the conclusions drawn adequately supported by the results?

Yes

Are sufficient details of methods and analysis provided to allow replication by others?

Yes

Reviewer Expertise:

Mixed methods research, health policy and systems research, implementation science

I confirm that I have read this submission and believe that I have an appropriate level of expertise to confirm that it is of an acceptable scientific standard, however I have significant reservations, as outlined above.

# Reviewer report: Reviewer response for version 2

**DOI:** 10.3310/nihropenres.14903.r32735

**Published:** 2024-09-18

**Authors:** Kathryn Almack, Jenni Lynch

**Affiliations:** 1 University of Hertfordshire, Hertfordshire, UK; 2 Centre for Research in Public Health and Community Care, School of Health and Social Work, University of Hertfordshire, Hatfield, England, UK

This paper contributes to discussions on the extent to which (and how) strengths-based practice (SBP) are drawn upon in practice and the challenges in doing so. As noted, there is little direct research evidence to support the effectiveness of SBP in the UK context. Given the existing reviews of this paper, my comments are focused on areas of interest which could be developed a little in this paper or in future papers and a couple of queries on the data collected to provide context.

 The study focuses on one region within the UK which has 14 Local Authorities. Would it be possible to have some further context in terms of what SBP approaches these Local Authorities were using? The results section discusses the Three Conversations model as one example, but it is not apparent if Local Authorities in the region adopt other models.

 Consulting with a panel of people with lived experience (who had either received adult social care services or had taken on a caring responsibility) and a stakeholder advisory group is an important aspect of this study. It might be helpful for other researchers to say a little about how the interview/survey results were presented to these groups to assist with the interpretation of findings – for example, what this a sense checking exercise?

 For further context, it would be useful to know workforce numbers of those working in or supporting ASC provision or in external organisations to assess the survey response rates i.e. what do the 286/131 responses represent as a percentage response rate?

 The authors discuss austerity (which might warrant a brief explanation for readers beyond the UK) as an external influence which can be both a driver and a barrier. Given that it is clearly stated in policy guidance that SBA approaches are not to be implemented as a cost saving measure, this is a complex issue since the ‘drivers’ do indeed appear to be to save costs by delaying input of ASC or length of time it may be required. There is a mismatch of perspectives about SBA implementation reported from various sources of data. The survey data from external organisations highlights the need For LAs to work more collaboratively with (and provide more support and resources for) external organisations, while the interview data with professional leaders presents a more positive picture. It would be interesting to unpack the complexity of this picture in more depth.

 The authors state that their data collection and analysis were guided by implementation theories (CFIR and NPT), but NPT is not explained in any depth other than key components of this framework being set out in a diagram. Did it play a part in the analysis? How were the two frameworks were combined; the headings of the Results section reflect the CFIR framework.

 Reported findings on SBP champions could be strengthened by providing additional evidence. The authors write that “A common approach to supporting implementation was training SBP champions. We noted a degree of inconsistency in their roles and responsibilities of champions”. How many of the 14 LAs had SBP champions? Given the importance of senior leadership noted elsewhere, were the SBP champion roles allocated to senior people? What were the roles and responsibilities of champions and thus the inconsistencies?

 Overall, research of this nature is invaluable in adding to the evidence base of the implementation of SBP although it also highlights there is scope for further research to fully unpack and understand the complexities involved.

Is the work clearly and accurately presented and does it cite the current literature?

Yes

If applicable, is the statistical analysis and its interpretation appropriate?

Not applicable

Are all the source data underlying the results available to ensure full reproducibility?

Yes

Is the study design appropriate and is the work technically sound?

Yes

Are the conclusions drawn adequately supported by the results?

Partly

Are sufficient details of methods and analysis provided to allow replication by others?

Partly

Reviewer Expertise:

Social care capacity building; adult social care research; intervention frameworks/theories; strength based approaches; mixed methods ; knowledge mobilisation

We confirm that we have read this submission and believe that we have an appropriate level of expertise to confirm that it is of an acceptable scientific standard, however we have significant reservations, as outlined above.

# Reviewer report: Reviewer response for version 2

**DOI:** 10.3310/nihropenres.14903.r32636

**Published:** 2024-09-13

**Authors:** Yu-Lun Chen

**Affiliations:** 1 Physical Medicine and Rehabilitation, Rutgers New Jersey Medical School, Newark, New Jersey, USA; 2 Center for Outcomes and Assessment Research, Kessler Foundation, East Hanover, New Jersey, USA

Thank you for the opportunity to review the revision. The authors have added more information on the definition of adult social care and the research method, which has substantially improved the manuscript. I also appreciated the diagram outlining the study in “Table 1,” although it should be labeled as a figure instead of a table.

 However, the figures (3-6) did not seem to improve in this revision. The graphs remain blurry and hard to read on the HIHR Open Research platform and in the downloaded PDF. Figure 3, as mentioned in the first review, is hard to interpret and would be better presented as a table (with rows representing the questions and columns showing the percentage of each response category) or as a horizontal bar graph with stacked columns. I will leave the decision on whether these issues need to be addressed to the editor and the authors.

Is the work clearly and accurately presented and does it cite the current literature?

Yes

If applicable, is the statistical analysis and its interpretation appropriate?

Not applicable

Are all the source data underlying the results available to ensure full reproducibility?

Yes

Is the study design appropriate and is the work technically sound?

Yes

Are the conclusions drawn adequately supported by the results?

Yes

Are sufficient details of methods and analysis provided to allow replication by others?

Partly

Reviewer Expertise:

Mixed-methods research, strength-based practice, occupational therapy

I confirm that I have read this submission and believe that I have an appropriate level of expertise to confirm that it is of an acceptable scientific standard.

# Reviewer report: Reviewer response for version 1

**DOI:** 10.3310/nihropenres.14688.r31963

**Published:** 2024-06-27

**Authors:** Yu-Lun Chen

**Affiliations:** 1 Physical Medicine and Rehabilitation, Rutgers New Jersey Medical School, Newark, New Jersey, USA; 2 Center for Outcomes and Assessment Research, Kessler Foundation, East Hanover, New Jersey, USA

Thank you for the opportunity to review this manuscript. This study evaluated the implementation of Strengths-Based Practice (SBP) in adult social care in England, using mixed methods incorporating data from interviews and two surveys. The study utilized two implementation frameworks, namely the Consolidated Framework for Implementation Research (CFIR) and Normalisation Process Theory (NPT), to guide the data collection and analysis. The research findings have valuable contributions to the adoption and challenges of SBP, informing the advancement of adult social care practices and related policies. I enjoyed reading this well-written manuscript. I would like to recommend a few minor changes to enhance the delivery of the research findings for readers from diverse backgrounds.

1. Introduction:
  - The authors may consider adding a brief description of adult social care defining the scope of services and related sectors/programs. This would help readers from other backgrounds better understand the topic, given that NIHR Open Research faces a diverse audience.
  - I appreciate the discussion of the varied definitions and common principles of SBP, which is instrumental for readers and clinical practitioners seeking to adopt SBP.
2. Methods:
  - I recommend adding an overview section (and/or diagram) explicitly describing or summarizing the research design, which will help a reader quickly understand the methods and the expected data elements. Given the large scope of the study, it would be helpful to outline the three data collection activities (the interviews and two surveys), including the distinct goal, timeline, and participants of each phase. It’d also be helpful to indicate when and how the two implementation frameworks were used, as well as when and how the lived experience/stakeholder advisory groups were involved.
  - “We adopted a mixed methods research design that included a scoping review of evidence, interviews with practice leaders and surveys with adult social care staff and external organisations in the region”: while this summary is helpful, it wasn’t explicitly clear to me what mixed methods design the authors used. I assume it was a Convergent Mixed Methods Design, where the qualitative and quantitative data were collected in a parallel timeline but not sequentially. However, it wasn’t clear whether the qualitative research guided the survey development, which would make it an Exploratory Sequential Design. It’d be helpful to clarify the design to enhance replicability.
  - “The survey data was mapped against insights from the interviews to examine any congruence or disparities in opinions between respondents”: Similar to the above, it’d be helpful to further elaborate on how the qualitative and quantitative research were integrated. It seems to me that the interviews were analyzed based on CFIR constructs, while the surveys were developed following NPT. Could you elaborate on how you compare the qualitative and quantitative findings? Did either qualitative or quantitative data collection influence the other?
3. Results
  - I appreciate that the authors integrated the qualitative and quantitative findings, structured using the CFIR. The findings were coherent and engaging.
  - However, I recommend the following to enhance the effective communication of the quantitative findings.
  - First, figures of the survey data are currently presented in low definition, and the text is hard to read. I strongly recommend the authors address this, such as by exporting the figures in higher definition (or in vector file format) or recreating them. Some figures may also be improved for data presentation. For example, Figure 3 might be better presented as a table, where the counts and percentages of each response category are compared side-by-side. Figures 4 & 5 could be enhanced with a label of percentages of each response category. Figure 6 seems to be a multiple-choice question, and if so, it’d be helpful to indicate this in the caption.
  - The authors may also consider consolidating survey findings with similar response categories in a table so that a reader can compare the percentage of each response category across questions. This is optional but will allow the authors to present more survey findings in addition to the selected questions.
  - Lastly, I would appreciate more descriptions of the samples of the interviews and the two surveys. For instance, for the interview participants, could you briefly describe the types of local authorities involved? For the surveys, it’d be helpful to include a table of participant characteristics including types of organizations, demographics, specialty areas, and professional experience level. I understand that some of this information is included in the extended data, however, summarizing this information in the paper would help a reader understand the context of the study and where the data came from.
4. Discussion:
  - The discussion is very insightful and provides actionable recommendations to improve the implementation of SBP.
  - Are there conflicting findings between the qualitative and quantitative data, or between the two surveys? It’d be helpful to discuss how these varied data sources complement and triangulate each other if space permits.

Is the work clearly and accurately presented and does it cite the current literature?

Yes

If applicable, is the statistical analysis and its interpretation appropriate?

Not applicable

Are all the source data underlying the results available to ensure full reproducibility?

Yes

Is the study design appropriate and is the work technically sound?

Yes

Are the conclusions drawn adequately supported by the results?

Yes

Are sufficient details of methods and analysis provided to allow replication by others?

Partly

Reviewer Expertise:

Mixed-methods research, strength-based practice, occupational therapy

I confirm that I have read this submission and believe that I have an appropriate level of expertise to confirm that it is of an acceptable scientific standard, however I have significant reservations, as outlined above.

# Reviewer report: Reviewer response for version 1

**DOI:** 10.3310/nihropenres.14688.r31971

**Published:** 2024-06-20

**Authors:** Madeleine Stevens

**Affiliations:** 1 London School of Economic and Political Science, London, UK

I thank the authors for this valuable research contribution on the topic of ‘strengths-based practice’. While the topic is widely discussed in relation to social care practice there is still little evidence of effectiveness, or of what strengths-based approaches (SBA) looks like in practice. This research aimed to explore perspectives on efforts to implement strengths-based practice in line with the UK Care Act in one UK region consisting of 14 local authorities. The researchers used interviews with people in the adult social care workforce, and two surveys, one with social care workforce and one with external organisations including independent and voluntary sector providers. This dual perspective is important given the aims of strengths-based approaches to better connect people with their communities.

 The researchers obtained impressive response rates in terms of principle social worker and lead occupational therapist interviewees. Again, including perspectives from both professions is crucial to the paper’s contribution.

 Survey data has been made available via links at the bottom of the article. Future researchers may want to request copies of the survey tools, which were based on existing frameworks for understanding implementation and normalisation but, as I understood it, developed specifically for this study. I wondered whether the authors might want to add descriptive statistics to give a little more information about the samples responding to the two surveys? For example, including the job roles of those responding?

 The findings align closely with our own research on one of the best-known of the ‘commissioned’ approaches, discussed here, the Three Conversations, which I mention particularly as it was published since the current paper was submitted (reference below).

 My comments are mainly around where more information would be of interest (but not essential) and where perhaps more circumspection in evidence claims might be useful i.e. perhaps a little more caution in the way some findings are expressed, or otherwise, more explanation of the strength of evidence.

 The authors may not think it appropriate to add more detail in the paper, or may not have this detail, but there are several important findings which would warrant further explanation. For example: it would be useful to understand more about the ways in which paperwork and recording systems create barriers, and constrain care pathways; why was 3Cs not seen as relevant to some local contexts (p7); why are SBA seen as less appropriate for more complex cases? It is reported that nearly 50% of adult social care staff respondents did not agree that their organisational structure supports strengths-based practice, though it should be noted that the large majority of these chose the ‘neither agree nor disagree’ option. As the site of potential barriers to effective practice, this is a finding worthy of future exploration.

 There are helpful explorations of other factors, for example of practitioners’ perceptions of why some people do not engage with  SBA, including that they fear engagement with family & community-based resources may lead to removal of existing care packages. In the paper this is related to insufficient explanation to, or understanding from people, about SBA. But, is this also a well-grounded fear? It’s one we also heard from people seeking support, in the paper referenced below.

 The evidence presented illustrates the differences of opinion, and interpretation, in relation to these approaches. As there are multiple sources of data it is occasionally not clear where the evidence comes from e.g. what was the evidence, or strength of evidence, in relation to lack of benefit of 3Cs over other approaches (a potentially important finding, but is the evidence sufficient for the claim?); On the other hand, use of consultancies 'appeared to have provided a more coherent plan and structure for implementation', again, what is the strength of evidence, and does this finding to some degree contradict the previous one on 3Cs?; How clear is the evidence supporting the claim that there were 'no well-defined and structured implementation plans' or that sites have 'rarely explicit plans' for implementation. There is some suggestion that it might be that this information was difficult to obtain rather than not existing?

 Lack of senior support, as in our research, is here linked to lack of effective implementation of strengths-based practice; perhaps what is next needed, to understand the benefits of SBA, is evidence as to how those LAs lose out compared to others. Other important findings include that bigger implementation projects drew in a greater variety of professionals which meant access to greater range of expertise and increased chances of success. With the same caveats needed in terms of the strength of evidence, limited involvement in developing and instigating implementation is, importantly, linked to poorer outcomes.

 There are plenty of positive views of practice with SBA referred to in the paper. The set of enablers on p12 is useful and fits with our findings, but we also found that while some of these steps (e.g. new forms and quality assurance frameworks) are designed to improve practice they don't always end up doing so (e.g. double paperwork) or are dropped – was this also found here? The enabler of reflective supervision is of interest, also peer support, though this seems to only be referred to in the discussion? I wonder if senior leadership support necessarily has to be associated with a top-down approach, as the authors imply in the discussion - could this not be part of an approach co-developed with staff at all levels, inside and outside ASC as well as with community members? Perhaps the authors' highlighting of the benefits of reflective supervision and peer support point towards this conclusion. The authors make useful suggestions for involving communities more, and refer to this helping identify barriers to delivery and innovation of services.

 Minor suggestions:

 In the paragraph beginning ‘Intervention characteristics’ p5, I’d suggest putting the framework components in inverted commas to make it easier to read.

 There are a few typos.

 Total number of respondents on bar charts would help with interpretation - e.g. could respondents choose as many options as they want?

 Reference mentioned above:[1]

Is the work clearly and accurately presented and does it cite the current literature?

Yes

If applicable, is the statistical analysis and its interpretation appropriate?

Not applicable

Are all the source data underlying the results available to ensure full reproducibility?

Yes

Is the study design appropriate and is the work technically sound?

Yes

Are the conclusions drawn adequately supported by the results?

Partly

Are sufficient details of methods and analysis provided to allow replication by others?

Partly

Reviewer Expertise:

Understanding and evaluating social care and mental health services for adults, children, and young people; community and family support; unpaid care; strengths- and relationships-based approaches; social determinants of health and wellbeing. Mixed methods research approaches.

I confirm that I have read this submission and believe that I have an appropriate level of expertise to confirm that it is of an acceptable scientific standard.

## References

[1] : Supporting Meaningful Implementation and Evaluation of Strengths-Based Approaches in Adult Social Care: A Theory of Change for The Three Conversations. *The British Journal of Social Work* .2024; 10.1093/bjsw/bcae055 10.1093/bjsw/bcae055
